# Supplementary material for: Microwave-field quantum metrology with inherent robustness against detection losses enabled by Rydberg interactions
Source: Nat Commun. 2026 Jul 24;17:9013. doi: 10.1038/s41467-026-76016-2 (PMC13507202; doi:10.1038/s41467-026-76016-2)
Supplement: Supplementary file 1 [file 41467_2026_76016_MOESM1_ESM.pdf]

Microwave-field quantum metrology with inherent robustness against detection losses enabled by Rydberg interactions

Stanisław Kurzyna,[1,2,*] Bartosz Niewelt,[1,2,*] Mateusz Mazelanik,[1] Wojciech Wasilewski,[1,2] Rafał Demkowicz-Dobrzański,[2] and Michał Parniak[1,2,†]

[1] *Centre for Quantum Optical Technologies, Centre of New Technologies, University of Warsaw, Banacha 2c, 02-097 Warsaw, Poland*

[2] *Faculty of Physics, University of Warsaw, Pasteura 5, 02-093 Warsaw, Poland*

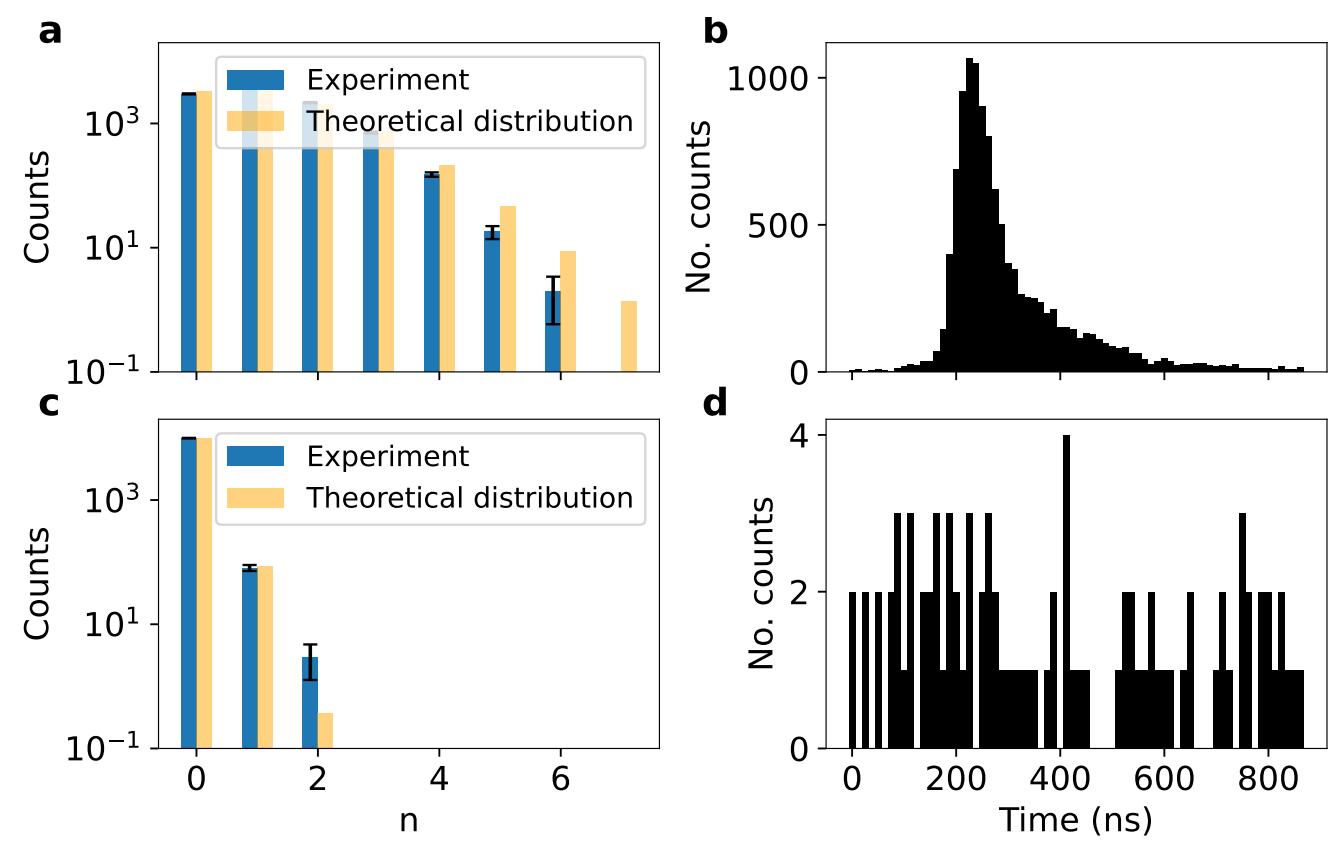

Supplementary Fig. 1. **Histograms of collected photons. a** Histogram of collected number of photons in the detection window with the added theoretical distribution of photons for the same mean number of detected photons. Error bars represent SE. **b** Binned photon counts measured in the read-out. **c** Histogram of dark counts in the detection window. The number of experimental sequences for both plots is $N = 10000$. Error bars represent SE. **d** Binned dark counts.

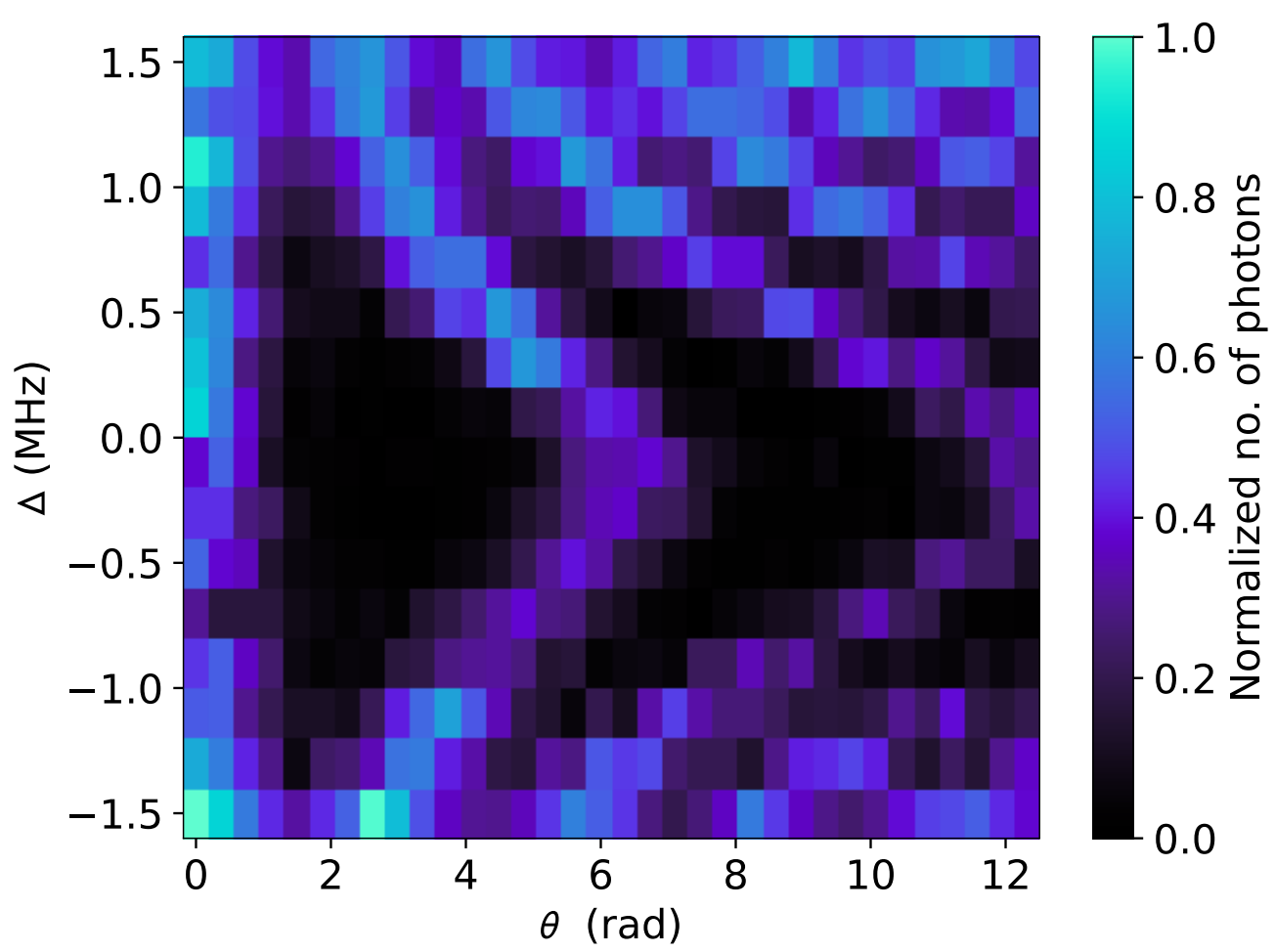

Supplementary Fig. 2. **Detuning dependence of the super Rabi oscillations.** Map of interaction-induced super Rabi oscillation between $|d\rangle$ and $|p\rangle$ for different detuning from the Rydberg transition resonance and super Rabi oscillation angle.

* Equal contributions

† michal.parniak@uw.edu.pl
