# Supplementary material for: Microwave-field quantum metrology with inherent robustness against detection losses enabled by Rydberg interactions
Source: Nat Commun. 2026 Jul 24;17:9013. doi: 10.1038/s41467-026-76016-2 (PMC13507202; doi:10.1038/s41467-026-76016-2)
Supplement: Supplementary file 2 [file 41467_2026_76016_MOESM2_ESM.pdf]

nature portfolio

Peer Review File

Microwave-field quantum metrology with inherent robustness against detection losses enabled by Rydberg interactions

Corresponding Author: Dr Michał Parniak

This file contains all reviewer reports in order by version, followed by all author rebuttals in order by version.

Attachments originally included by the reviewers as part of their assessment can be found at the end of this file.

Version 0:

Reviewer comments:

Reviewer #1

(Remarks to the Author)
In the manuscript titled "Microwave-field quantum metrology with error correction enabled by Rydberg atoms", the authors report an experimental demonstration of a quantum metrology protocol with cold Rydberg atoms that incorporates an error-correction like strategy to mitigate the effect of detection losses. The authors implement an effectively non-unitary operation via dipolar Rydberg interactions that removes the irreversible state component under loss. They show that this strategy is theoretical optimal in the two-excitation case. Using this method, they report an estimation precision corresponding to a microwave field sensitivity of 39 nV. cm^{-1} Hz^{-1/2}.

As a theorist, I will comment mainly on the theoretical contribution of this work and leave the experimental part for other referees. Overall, I think the proposed protocol is interesting and this work may potentially be suitable for publication as the potential broad applications of this sort of technique to enhance quantum sensing. However, I have some concerns as explained below:

1. It seems that the proposed protocol where a non-unitary operation is applied prior to a noisy measurement was already proposed before in this theory paper:

[*] “Optimal Protocols for Quantum Metrology with Noisy Measurements” (PRX Quantum 4 (4), 040305)

and the similarity between this one and the current manuscript should be clarified. For example, The “ultimate QFI” defined in the manuscript seems to be the same as the “preprocessing-optimized Fisher information” defined in Ref.[*]. The setting of this manuscript also seems to fit into the general scenario discussed in Sec.IVD of Ref.[*], and thus the analytical results in Ref.[*] should be compared to what’s obtained in this manuscript.

2. The proposed non-unitary operation should be called neither “error correction” nor “error mitigation”, both of which are mentioned many times in the manuscript to describe the procedure. First, there is no error correction in this procedure, which involves syndrome measurement first and decoding operation afterwards—which is why the procedure should not be called “error correction”. Second, error mitigation typically involves classical data processing procedure and noise learning procedure to extrapolate or recover the noiseless behavior of a quantum system, which seems to be quite different from what's done in this experiment. I suggest the authors use a different, more suitable terminology, for example, “preprocessing protocol” as proposed in Ref.[*] or “interaction-based readout” as used in other previous literature, e.g. PhysRevLett.119.193601, PhysRevA.98.030303, etc.

3. “Optimality of error mitigation” in Methods suggested the optimality of the proposed protocol is verified numerically, but I see no numerical data provided in the “DATA AVAILABILITY” section. Moreover, from Fig.1(d) it seems like the proposed procedure is only optimal in certain range of theta, then was the numerical algorithm performed also only at these optimal points of theta? Furthermore, what is the distinction between the iterative optimization described in Methods and the one proposed in Sec.III of Ref.[*]?

4. The Kraus operators are defined as K0=pd, K1=sqrt{1-p^+pd^+d}. I understand that this is well defined within the four

level system considered ({|2,0>,|1,1>,|0,2>,|0,0>}). However, in general, sqrt{1-p^+pd^+d} doesn't seem to be well defined as 1-p^+pd^+d can certainly be negative. Therefore, it is important to clarify what happens to other quantum states in the Hilbert space by your operation. Probably, a better and clearer way to write down your Kraus operator is K0=|0,0><1,1|, K1=|0,2><0,2|+|2,0><2,0|, K3= identity operation on all other levels. The terminology is also a bit confusing when you say your non-unitary operation "removes" the |1,1> state. As far as I can tell, it is not removed, and instead it just switches to the state |0,0>.

5. It seems that if you define a unitary operation U = |0,2><0,2|+|2,0><2,0|+|0,0><1,1|, the performance of your Fisher information should be the same as your current protocol because all off-diagonal terms like |2,0><0,0| in your density matrix effectively do not contribute to your detection event. Could the author confirm whether this is the case? Then is it true that the reason of implementing a non-unitary operation in your scenario is because the non-unitary operation is easier to implement than unitary operations in the experiment?

Reviewer #2

(Remarks to the Author)
I co-reviewed this manuscript with one of the reviewers who provided the listed reports. This is part of the Nature Communications initiative to facilitate training in peer review and to provide appropriate recognition for Early Career Researchers who co-review manuscripts.

Reviewer #3

(Remarks to the Author)
The manuscript presents an interesting idea for surmounting measurement noise in quantum metrology: perform a nonunitary operation on the quantum state to surmount inefficient detectors. This type of strategy has been studied before in terms of placing filters before detectors, which is the realm of postselected metrology that has advantages with detectors that saturate, and in seeded nonlinear interferometry, where amplification overcomes detector inefficiencies. The difference here is that the operations are probability-preserving (no states need be discarded with a filter) and that loss is used instead of amplification (which is apparently optimal for a toy model). An experimental realization with Rydberg atoms is then presented.

Unfortunately, while the toy model is compelling, its generalization to further classes of input states is lacking and the experimental demonstration seems very different from the theoretical model. The ultimate bounds that are overcome here are also not rigorously defined (in terms of different input states, detector inefficiencies, and per-photon vs aggregate results). For these reasons I cannot recommend publication in Nature Communications. Below I evidence these points and state further questions, roughly in the order of appearance in the manuscript.

1. The beginning of the manuscript introduces a bound to beat, given by the efficiency times the QFI. This bound is not justified, other than "typical situations" and "detection losses act independently on each sensing particle." I do not see from whence this arises and this is a bit of a non-starter for my review. Upon reading Ref. [38], I find that QFI is always reduced by some factor, as one would expect, but I do not find that QFI is always reduced by this factor of detection efficiency. I can imagine a NOON state for interferometry subject to detector inefficiencies eta, where the performance is much worse: |N0>+exp(i phi)|0N> decays to the same state with probability eta^N, in convex combination with states |k0><k0| + |0k><0k| with probabilities binom(N,k) eta^k (1-eta)^(N-k); the QFI goes down by a factor of eta^N << eta because the rest of the states in the convex combination are independent from the parameter phi being probed. Clearly the bound presented here works for coherent states in that scenario whose amplitude is simply diminished by eta, but more justification is required for this "typical" bound and its universality if the goal here is to beat it. Perhaps this is a universal upper bound and the NOON state example respects that, even if said upper bound is not attainable there, so that beating this upper bound is useful here?

2. One impressive aspect of these results is that they are not postselective. The nonlinear loss channel introduced here does not throw away any probability but simply transforms the state so that it is always sent to the detectors. Specifically, ambiguous detection events are always converted into |00> so that they are removed from erroneously adding to the "signal." In some sense, though, could this be cast as postselected metrology? If the detection events are all about counting excitations and the |11> state is filtered out so that it produces no detection events, it seems highly related.

3. Another avenue that has been explored for overcoming detector inefficiencies in interferometry is seeding, a sort of nonlinear amplification instead of nonlinear loss. Would that work here as well? If the states |20> and |02> are amplified in the toy model, would they help overcome loss? Or is the point that all amplification also adds noise and that's why strategies adding loss could be ideal?

4. When discussing Fig. 1(d) in the text, the authors state that there is an increased QFI due to the nonunitary operation Lambda. But that is impossible. I presume they meant that there is an increase in Fisher information for a particular measurement strategy? Otherwise something is breaking down here, as QFI is nonincreasing under quantum operations.

5. When I get to the experiment, I worry that it does something rather different than that proposed in the theory. The theory proposes that |11> goes to |00>, while the experiment says that |11> dephases with the rest of the system, but no excitations are lost. In that case, I do not understand how any protection is granted against detection inefficiencies.

6. Continuing to the description of super Rabi oscillations, I again fear that the dynamics are far from what was described in the theory. It now appears (5) that the number of excitations in each of the modes decays exponentially due to the

interactions, whereas we wanted only the terms with excitations of both modes to decay. The former decays to zero while the latter decays to some constant given by the probability that there are excitations in only one mode. In this case I really do not understand why one desires tau to be "the longest possible" if it means that all of the excitations fade away---it seems that there the signal would be completely lost!

7. As well, the super Rabi oscillation method goes in the face of the proposed theory when no correlations or entanglement arises. The theory would say "start with a bi-coherent state and drop all terms in the superposition where there are excitations in both modes. Then you will be left with exp(-n sin^2(t))*|sqrt(n)cos(t),0> + exp(-n cos^2(t))*|0,sqrt(n)sin(t)> in convex combination with zero-excitation terms." The first term clearly looks entangled and correlated between the two modes.

8. What are CT beams here? Computed Tomography? Much of Fig. 3 is not explained in the main text but perhaps it is in the methods section. I see that it is, but the main text is rather confusing on its own.

9. What is "the probability distribution of the mean number of detected photons?" Is it the distribution of the number of photons, or is it actually the distribution of the mean? Anyway, I assume it's the probability distribution for the number of photons; in which mode, then? Is it the two-mode joint distribution or the one-mode marginal distribution? Again I see an answer in the methods but the main text is confusing on its own.

10. What is the expected advantage in FI given the toy model, assuming photon detection as the measuremet scheme? How does it compare to the 3.3 advantage here? Does the QFI indeed diminish by a factor of eta in this case, thereby justifying the normalization used to compute the 3.3 advantage? Otherwise unfortunately most of these comparisons are meaningless.

11. What do the numbers mean when it says there is an average of 1.1 photons per pulse, the uncertainties are shown per 100 detected photons, and the estimation precision is per experimental shot; does this mean the precision of Delta E_MW is given per 100/1.1=91 pulses? Is this factored into the duty cycle calculation, then, by assuming some repetition rate of the pulses?

12. Now we get back to Fisher information per detected photon. This is actually the realm of postselected metrology and needs to be appropriately quantified. If you triple the FI/photon and reduce the probability of detecting a photon by 3 then you have not achieved anything overall, except in the case of detector saturation. So the advantage in the experiment is certainly not obvious.

13. Getting to the methods, this does not seem to be sufficient for reproduceability. The authors claim that they used some optimization strategy defined in their previous work and then the numerically found optimal solution coincides with their toy model. The reader is expected to take all of this on faith and then also believe that the numerical solution is therefore the global optimum. It may all be true, but as a reviewer I cannot accept any of it. There isn't even some analytical evidence that the current strategy is a local optimum of any sort.

14. Then in the methods it appears that a single-photon counting module is used; again, this looks different from the theory of looking at the full photon number distribution or at states like |20> and |02>.

15. Still in the methods, now it looks as though the mean photon number in mode d is actually just computed as the mean photon number in mode d that arises from the |1,1> state. This is quite different from what was described in the main text. As well, nothing is given to explain how this evolution affects the other states, especially given that we have a pair of weak coherent states.

16. Finally, the phenomenological model for the photon statistics is fine, but I am not sure how seriously I should believe that it actually models the evolution (except perhaps in the case of very weak coherent states?).

Version 1:

Reviewer comments:

Reviewer #2

(Remarks to the Author)
I co-reviewed this manuscript with one of the reviewers who provided the listed reports. This is part of the Nature Communications initiative to facilitate training in peer review and to provide appropriate recognition for Early Career Researchers who co-review manuscripts.

(Remarks on code availability)
I have able to install and run the numerical analysis code related to the optimality of the pre-measurement processing protocol. The authors have provided clear and well-documented instructions for installing and executing the code.

Reviewer #3

(Remarks to the Author)
In the response letter by the authors, they "stress again that the main contribution of this paper is experimental and not

theoretical, and the theory part served merely as a means to understand the naturally appearing `detection noise robustness' mechanism that we observed in the experiment." This complicates the question of whether the manuscript should be worthy of publication in Nature Communications, as the main work seemed to include much theory. One manner in which the authors have addressed this issue is by modifying the order of presentation so that the toy model appears last. This certainly helps centre the discussion on the experiment but I worry that it is a bait-and-switch tactic for the referees, who have mainly focused on the theory in the first round of review, and for the editors in prompting them to select theorists as reviewers.

Overall, I think it is nice to have an experiment showcasing that a state may be modified prior to an inefficient detector in order to restore some of the performance of the detector. I am not convinced that this method is general for overcoming loss in arbitrary platforms with arbitrary input states, but this demonstration is meritorious. I no longer feel strongly that this manuscript should be rejected from Nature Communications and am rather neutral about whether the experiment itself is worthy of publication there, with a slight leaning toward recommending publication due to this being am important theory (not novel here) that has not yet been demonstrated (to the best of my knowledge).

In response to the responses to referee #3, there are two outstanding points:
1. The language in the introduction is much more accurate and I appreciate the clarifications. However, it still just states that the QFI diminishes linearly in typical situations and does not provide justification/citation, which I feel may be necessary when this is the major hurdle surmounted by this work. Otherwise it is a straw man argument.

2. I cannot find where the authors included non-linear amplification. Is this supposed to be subsumed by the Gamma in the Methods section Eq. (38)? The response 3 says that the scaling with losses would be "even more favourable" but the section surrounding Eq. (38) says that the present method is already optimal. What is the true result?

(Remarks on code availability)

Reviewer #4

(Remarks to the Author)
Please see the "Review Attachments" file.

(Remarks on code availability)

Version 2:

Reviewer comments:

Reviewer #3

(Remarks to the Author)
The authors have adequately addressed most of my queries. I am not certain why most of the amplification material is in a python script in the attached dataset and believe that explaining this in more detail in the main text would be reasonable.

(Remarks on code availability)
n/a

Reviewer #4

(Remarks to the Author)
See the attached PDF file.

(Remarks on code availability)

Open Access This Peer Review File is licensed under a Creative Commons Attribution 4.0 International License, which permits use, sharing, adaptation, distribution and reproduction in any medium or format, as long as you give appropriate credit to the original author(s) and the source, provide a link to the Creative Commons license, and indicate if changes were made.
In cases where reviewers are anonymous, credit should be given to 'Anonymous Referee' and the source.
The images or other third party material in this Peer Review File are included in the article's Creative Commons license, unless indicated otherwise in a credit line to the material. If material is not included in the article's Creative Commons license and your intended use is not permitted by statutory regulation or exceeds the permitted use, you will need to obtain permission directly from the copyright holder.
To view a copy of this license, visit https://creativecommons.org/licenses/by/4.0/

Reviewers' comments and authors' response

Reviewer #1 (Remarks to the Author):

Comment 0
In the manuscript titled "Microwave-field quantum metrology with error correction enabled by Rydberg atoms", the authors report an experimental demonstration of a quantum metrology protocol with cold Rydberg atoms that incorporates an error-correction like strategy to mitigate the effect of detection losses. The authors implement an effectively non-unitary operation via dipolar Rydberg interactions that removes the irreversible state component under loss. They As a theorist, I will comment mainly on the theoretical contribution of this work and leave the experimental part for other referees. Overall, I think the proposed protocol is interesting and this work may potentially be suitable for publication as the potential broad applications of this sort of technique to enhance quantum sensing. However, I have some concerns as explained below:

Response 0
We would like to thank the Reviewer for a thorough reading of our manuscript and the constructive remarks, which we address point by point below. We are also thankful for finding our manuscript worthy of publication in Nature Communications.

Comment 1
1. It seems that the proposed protocol where a non-unitary operation is applied prior to a noisy measurement was already proposed before in this theory paper:

[*] “Optimal Protocols for Quantum Metrology with Noisy Measurements” (PRX Quantum 4 (4), 040305)show that this strategy is theoretical optimal in the two-excitation case. Using this method, they report an estimation precision corresponding to a microwave field sensitivity of 39 nV. cm^{-1} Hz^{-1/2}.

and the similarity between this one and the current manuscript should be clarified. For example, The “ultimate QFI” defined in the manuscript seems to be the same as the “preprocessing-optimized Fisher information” defined in Ref.[*]. The setting of this manuscript also seems to fit into the general scenario discussed in Sec.IVD of Ref.[*], and thus the analytical results in Ref.[*] should be compared to what’s obtained in this manuscript.

Response 1
The Reviewer is perfectly correct. Indeed, the theoretical scenario we are considering is by no means original, and we referred in particular to paper [31], where one of the Authors is also the co-author of Ref [*]. While referring to [31], we have unfortunately missed the important Ref [*]. In fact, to be completely honest, shortly after submitting the paper, we were contacted by one of our colleagues (Janek Kolodynski) pointing out to the Ref [*] as an important follow-up to Ref.

[31], where a number of methods to confirm the optimality of the “measurement preprocessing” protocols have been provided. Irrespectively, of the reports we have received, we planned to incorporate this reference in any future resubmissions. We have now cited [*] as Ref. [38]. This said, we should stress again that the main contribution of this paper is experimental and not theoretical, and the theory part served merely as a means to understand the naturally appearing `detection noise robustness' mechanism that we observed in the experiment. Even though we have not used the superior methods of Ref [38], and instead applied a more universal framework of numerical quantum channel optimisation, which was more straightforward for us to employ, the numerical results we have obtained were credible enough to conjecture the optimality of the strategy. With the help of Ref [38], we are now able to confirm that the solution found is indeed optimal. Our case falls into the category of pure state and commuting POVMs case discussed in Sec IVD of Ref [38]. More concretely, we have checked that indeed formula (66) of Ref [38] yields the same reduction coefficient \gamma = (2-\eta) \eta, as we get at the operating point \theta=\pi/2, where the protocol is operating optimally. We have now significantly expanded the Method section, where we prove the optimality of the protocol using the above arguments, and also changed the unfortunate title of the section to “Optimality of premeasurement processing protocol”.

We are thankful to the Referee for bringing this issue up. We are convinced that now, this issue is fully resolved, and the presentation of the results in the paper has improved both in terms of rigour and clarity.

Comment 2
2. The proposed non-unitary operation should be called neither “error correction” nor “error mitigation”, both of which are mentioned many times in the manuscript to describe the procedure. First, there is no error correction in this procedure, which involves syndrome measurement first and decoding operation afterwards—which is why the procedure should not be called “error correction”. Second, error mitigation typically involves classical data processing procedure and noise learning procedure to extrapolate or recover the noiseless behavior of a quantum system, which seems to be quite different from what's done in this experiment. I suggest the authors use a different, more suitable terminology, for example, “preprocessing protocol” as proposed in Ref.[*] or “interaction-based readout” as used in other previous literature, e.g. PhysRevLett.119.193601, PhysRevA.98.030303, etc.

Response 2
We are sorry for the sloppiness in the nomenclature used. In fact, we have tried to avoid speaking of `quantum error-correction' for this very reason, and used a more ambiguous `error-correction' or `quantum error-correction inspired protocol' phrase. Still, we agree that this led to unnecessary confusion and went against the common nomenclature used in the field of quantum information. We have now decided to go for the “inherent robustness against detection losses” term. On one hand, this clearly points to the “pre-measurement stage” as the place where the information is transformed in a way that will be affected less by the measurement noise. Second, it indicates that we are not really by hand introducing any preprocessing operations, but rather observing that it happens naturally in the experimental setup we work with. In our opinion, this resolves the double confusion that might have arisen. One related to sloppy use of error-correction terminology, and the other one, related to the

perception of our work as an intentionally designed experiment to illustrate the “pre-processing optimised QFI” ideas. We hope that both sources of confusion are now eliminated.

Comment 3

3. “Optimality of error mitigation” in Methods suggested the optimality of the proposed protocol is verified numerically, but I see no numerical data provided in the “DATA AVAILABILITY” section. Moreover, from Fig.1(d) it seems like the proposed procedure is only optimal in certain range of theta, then was the numerical algorithm performed also only at these optimal points of theta? Furthermore, what is the distinction between the iterative optimization described in Methods and the one proposed in Sec.III of Ref.[*]?

Response 3

Thank you for noticing this issue. In the revised version of the article, we attached a link to the optimisation script. As you correctly noted, the operation is optimal only for some range of the parameter \theta. We optimized the QFI for a fixed parameter, and while the resulting channel is optimal for this value of \theta, the operation can be easily modified by adding a unitary rotation (similar to the parameter encoding) to be optimal in a different range of parameters.
This issue is now of lesser importance, though in light of a rigorous argument of the optimality of the protocol found, which is now explained in detail in the Methods section.

Regarding the differences between our iterative optimisation and the one from Sec.III of Ref [38], there are some subtle differences, which make the two approaches inequivalent. In Ref [38], the figure of merit is the classical FI under fixed measurement, where the premeasurement channel is optimized. In our case, we filter out the noisy part of the measurement, treating it as a fixed channel, and optimise the quantum FI of the output state of the protocol, optimized over the preprocessing channel. Hence, in our approach, we might in principle obtain higher quantum FI, as we do not fix the measurement. This reflects, the overall character of the QMetro++ package we have used, which is thought of as a quantum FI optimisation package. Still, in the problem considered, the results of the optimisation are identical, since the natural measurement in the atom-occupation basis is optimal. The other formal distinction is the formulation of the problem in Ref [38] as minimisation of the inverse of classical FI, while we write it as a maximisation of the quantum FI. These, however, do not introduce any additional aspects that would make the approaches inequivalent (it could be relevant in the multi-parameter estimation case, though).

Comment 4

4. The Kraus operators are defined as K0=pd, K1=sqrt{1-p^+pd^+d}. I understand that this is well defined within the four level system considered ({|2,0>,|1,1>,|0,2>,|0,0>}). However, in general, sqrt{1-p^+pd^+d} doesn’t seem to be well defined as 1-p^+pd^+d can certainly be negative. Therefore, it is important to clarify what happens to other quantum states in the Hilbert space by your operation. Probably, a better and clearer way to write down your Kraus operator is K0=|0,0><1,1|, K1=|0,2><0,2|+|2,0><2,0|, K3= identity operation on all other levels. The terminology is also a bit confusing when you say your non-unitary operation “removes” the |1,1> state. As far as I can tell, it is not removed, and instead it just switches to the state |0,0>.

Response 4

Thank you for this important comment. As you correctly pointed out, the proposed Kraus operators are only valid when we are considering the Hilbert space limited to at most two excitations. We chose this representation as we think it is easier to intuitively understand the operation performed by this quantum channel, and more importantly, it is easier to see the connection between dipolar Rydberg-Rydberg interactions and the proposed form of the Kraus operators. We agree with the Reviewer, as to the bit confusing representation of the operators. Following the Reviewer's suggestion, we changed the Kraus operators in the article and corrected the confusing terminology. Additionally, we included a part in "Methods" that explains the connection between the toy and the multi-particle models (previously phenomenological). We have also given additional argumentation for the physical underpinnings of the four-level system Kraus operators, and we also demonstrated the backwards-compatibility of these operators with the more general case given by Eq. (28) of the revised manuscript.

Comment 5

5. It seems that if you define a unitary operation U = |0,2><0,2|+|2,0><2,0|+|0,0><1,1|, the performance of your Fisher information should be the same as your current protocol because all off-diagonal terms like |2,0><0,0| in your density matrix effectively do not contribute to your detection event. Could the author confirm whether this is the case? Then is it true that the reason of implementing a non-unitary operation in your scenario is because the non-unitary operation is easier to implement than unitary operations in the experiment?

Response 5

Thank you for this thoughtful insight. The unitary operation you proposed is also optimal, and results in increased QFI, in the same way as the operation presented in the article. We should, however, stress again that we are NOT designing the preprocessing operation for our protocol, but just interpret and show the optimality of the process that appears naturally thanks to the intrinsic and controllable Rydberg-Rydberg dipolar interactions. Hence, the non-unitary operation discussed in the article is what we face in our experimental system, and there is no point in looking for an equivalent unitary operation that might perform as well as this one, but would require additional highly non-trivial (if at all possible) experimental effort. Our revised manuscript has been reformulated to stress the physical and experimental foundation rather than only the quantum-information-theoretic aspects, in order to reflect this. We hope that this reformulation, along with the refined proof of the optimality of our operation (in the reduced, toy-model space), will resolve this issue.

Reviewer #2 (Remarks to the Author):

I co-reviewed this manuscript with one of the Reviewers who provided the listed reports. This is part of the Nature Communications initiative to facilitate training in peer review and to provide appropriate recognition for Early Career Researchers who co-review manuscripts.

Reviewer #3 (Remarks to the Author):

Comment 0

The manuscript presents an interesting idea for surmounting measurement noise in quantum metrology: perform a nonunitary operation on the quantum state to surmount inefficient

detectors. This type of strategy has been studied before in terms of placing filters before detectors, which is the realm of postselected metrology that has advantages with detectors that saturate, and in seeded nonlinear interferometry, where amplification overcomes detector inefficiencies. The difference here is that the operations are probability-preserving (no states need be discarded with a filter) and that loss is used instead of amplification (which is apparently optimal for a toy model). An experimental realization with Rydberg atoms is then presented.

Unfortunately, while the toy model is compelling, its generalization to further classes of input states is lacking and the experimental demonstration seems very different from the theoretical model. The ultimate bounds that are overcome here are also not rigorously defined (in terms of different input states, detector inefficiencies, and per-photon vs aggregate results). For these reasons I cannot recommend publication in Nature Communications. Below I evidence these points and state further questions, roughly in the order of appearance in the manuscript.

Response 0
We would like to thank the Reviewer for a thorough reading of our manuscript and the constructive remarks, which we address point by point below.

Comment 1
1. The beginning of the manuscript introduces a bound to beat, given by the efficiency times the QFI. This bound is not justified, other than "typical situations" and "detection losses act independently on each sensing particle." I do not see from whence this arises and this is a bit of a non-starter for my review. Upon reading Ref. [38], I find that QFI is always reduced by some factor, as one would expect, but I do not find that QFI is always reduced by this factor of detection efficiency. I can imagine a NOON state for interferometry subject to detector inefficiencies eta, where the performance is much worse: |N0>+exp(i phi)|0N> decays to the same state with probability eta^N, in convex combination with states |k0><k0| + |0k><0k| with probabilities binom(N,k) eta^k (1-eta)^(N-k); the QFI goes down by a factor of eta^N << eta because the rest of the states in the convex combination are independent from the parameter phi being probed. Clearly the bound presented here works for coherent states in that scenario whose amplitude is simply diminished by eta, but more justification is required for this "typical" bound and its universality if the goal here is to beat it. Perhaps this is a universal upper bound and the NOON state example respects that, even if said upper bound is not attainable there, so that beating this upper bound is useful here?

Response 1
We thank you for highlighting this point, which we now realise may indeed be confusing without specifying more explicitly the meaning of the term “typical situations”. Clearly, the \eta F_Q formula is strictly valid for systems of independent probes, or approximately for weakly correlated probe states. For correlated systems, it may deteriorate more rapidly (as indicated by the Referee using the NOON state example), or weaker (as is the case for the preprocessed state, which is the focus of this work). However, in practical sensing experiments, correlated particles are not used, and information scales linearly with losses. While, of course, the field of quantum metrology explores those “other” situations, the use of uncorrelated particles is vastly more common in experiments and engineering.

We have modified this part of the Introduction accordingly, to avoid any unjustified claims and kept the main message that is that the protocol considered may be understood in the spirit of replacing effectively uncorrelated probes (in this case, atomic excitations) with a correlated state that preserves parameter information in a more robust way in the presence of detection losses.

Comment 2

2. One impressive aspect of these results is that they are not postselective. The nonlinear loss channel introduced here does not throw away any probability but simply transforms the state so that it is always sent to the detectors. Specifically, ambiguous detection events are always converted into |00> so that they are removed from erroneously adding to the "signal." In some sense, though, could this be cast as postselected metrology? If the detection events are all about counting excitations and the |11> state is filtered out so that it produces no detection events, it seems highly related.

Response 2

Indeed, as the Referee correctly points out, we are NOT following here the post-selection paradigm. If we wanted to follow it, we would rather discard the |1,1> component and renormalise the remaining state. This would further increase the resulting QFI, as is typically the case in the post-selection metrology paradigm (at least in the \theta region around the optimal operating point \pi/2). In our case, we include this contribution, which in the end leads to no detection events and lowers the observed total excitation numbers. Still, despite this more honest approach, we are able to demonstrate the advantage over the “no-preprocessing case”. We added a comment on this in the text (last paragraph of “Toy Model”) to clearly distinguish our approach from a post-selection metrology one.

Comment 3

3. Another avenue that has been explored for overcoming detector inefficiencies in interferometry is seeding, a sort of nonlinear amplification instead of nonlinear loss. Would that work here as well? If the states |20> and |02> are amplified in the toy model, would they help overcome loss? Or is the point that all amplification also adds noise and that's why strategies adding loss could be ideal?

Response 3

Thank you for the thoughtful insight. In fact, if we applied a non-linear amplification such that state |2,0> -> |N,0> and |0,2> -> |0,N> we would also observe an even more resilient response against detection losses. Still, the FI would not exceed the initial value, but scaling with losses would be even more favourable. We attached a script with a numerical evolution of the state under the operation proposed in the article. Additionally, we included a part devoted to testing the mentioned amplification scheme. Such an amplification operation is not experimentally feasible at the moment, but is certainly of great interest for future work.

Comment 4

4. When discussing Fig. 1(d) in the text, the authors state that there is an increased QFI due to the nonunitary operation Lambda. But that is impossible. I presume they meant that there is an increase in Fisher information for a particular measurement strategy? Otherwise something is breaking down here, as QFI is nonincreasing under quantum operations.

Response 4

Thank you for stating this important question. As you correctly observed, the increase in FI manifests when comparing to detection without interactions. This means that it does not exceed the initial value of QFI without losses. We have now explained it more clearly in the text (last but one paragraph of Toy Model).

Comment 5

5. When I get to the experiment, I worry that it does something rather different than that proposed in the theory. The theory proposes that |11> goes to |00>, while the experiment says that |11> dephases with the rest of the system, but no excitations are lost. In that case, I do not understand how any protection is granted against detection inefficiencies.

Response 5

Thank you for the important remark. The presented toy model is merely a simple model to intuitively understand a method to increase the FI for this measurement strategy. We do not claim that it fully corresponds to the experimental setting. The Reviewer rightly noted that the dephased excitations are not the same as losing the excitation. But there are some subtleties that we need to address. By dephased excitation, we meant that components of the spinwave, which contain delocalized excitations in both modes, will acquire additional phase (resulting from the interaction). These components will no longer fulfil the phase-matching condition, rendering them insensitive to the read-out. This means that the delocalized excitations will remain in the atomic cloud and affect the evolution; however, they will not be directly detectable. This is effectively the same as removing the state that contains excitations in both modes from the accessible system. The figures have also been refined to demonstrate this dephasing as a loss leading the excitations to different modes, outside of the scope of our detection scheme.

Comment 6

6. Continuing to the description of super Rabi oscillations, I again fear that the dynamics are far from what was described in the theory. It now appears (5) that the number of excitations in each of the modes decays exponentially due to the interactions, whereas we wanted only the terms with excitations of both modes to decay. The former decays to zero while the latter decays to some constant given by the probability that there are excitations in only one mode. In this case I really do not understand why one desires tau to be "the longest possible" if it means that all of the excitations fade away---it seems that there the signal would be completely lost!

Response 6

Indeed, this is an important remark. The previous Eq. (5) is a simplified version of Eq. (27) (previously Eq.(24)) with the assumption that the \gamma \tau << 1, which is applicable for our experimental parameters, where \gamma \tau ~=0.04. It is important to note that, in the explicit expression for the mean number of detected photons, for large values of \gamma \tau, the exponent is saturated, and the decay is proportional to the number of excitations in the opposite mode. Hence, for the given number of initial excitations stored in the memory, we want the interaction time to be the longest possible, allowing us to get closer to the saturation regime of the interaction-induced decay. This increases the steepness of the super Rabi oscillations, and thus increases the precision of the estimation and consequently the sensitivity of MW field detection. However, note that due to the finite lifetime of the stored excitation (~30us), we have

to balance \tau carefully, as increasing \gamma \tau results in steeper super Rabi oscillations but also reduces the number of observed photons. We found that in our case, \tau=20us is sufficient. We have amended the script to include this explanation and moved the explicit expression for expectation value from the methods to the end of the “Theoretical model” subsection in the main text to make it clearer.

Comment 7

7. As well, the super Rabi oscillation method goes in the face of the proposed theory when no correlations or entanglement arises. The theory would say "start with a bi-coherent state and drop all terms in the superposition where there are excitations in both modes. Then you will be left with exp(-n sin^2(t))*|sqrt(n)cos(t),0> + exp(-n cos^2(t))*|0,sqrt(n)sin(t)> in convex combination with zero-excitation terms." The first term clearly looks entangled and correlated between the two modes.

Response 7

Thank you for addressing this important point. As the reviewer correctly noticed, the presented state will be entangled. However, in this particular experimental scheme, we do not detect the correlations and the entanglement during the measurement process. Note that we do not need to detect it, as we show in the Toy Model that the optimal results can be achieved without measuring the correlations and entanglement between the states.

Additionally, to understand what happens with the state of the excitation, without the loss of generality, we can look only at the *d* mode. The collective excitations (Rydberg spin waves) in the mode *d* during the evolution will acquire the spatial phase, according to Eq. (16) (previously Eq.(14)) in the Methods section. The excitations are not getting lost due to dipolar interaction, since the evolution only appears as the phase component. A part of the spinwave is scattered into unobservable but still relevant modes with different values of **k**=/=0. This results in the observed interaction-induced decay. That is because the intensity of the light recovered from atoms by reversing the light-atoms interface is given by the number of excitations in spin wave with **k**=0. The scattered components will no longer fulfil the phase-matching condition and will not be efficiently read out from the memory. We amended the script to include this explanation (“Theoretical model” subsection).

We admit that the phrasing was unfortunate; we wanted to justify the simple model to calculate the base decay rate between two excitations and to extend it for multiple excitations. The many-body effects happen with considerably lower probability, and so we strongly believe that the extension of the model is justified. We edited this part of the article (the end of subsection “Theoretical model”) to convey this message more clearly. We now also show the full compatibility of the two-excitation toy model, the many-excitation model, and the effective result for the mean photon numbers. Therefore, we would like to stress that the observed mean values are fully in line with the complete many-body model.

Comment 8

8. What are CT beams here? Computed Tomography? Much of Fig. 3 is not explained in the main text but perhaps it is in the methods section. I see that it is, but the main text is rather confusing on its own.

Response 8

Thank you for noticing. By CT beams, we meant Coherence Transfer beams, which are the beams that remove the wavevector of the created Rydberg atomic coherence. This way, the stored coherence is transferred in such a way that it is less susceptible to the residual thermal motion of the atomic ensemble, allowing for a longer memory lifetime. We have amended the manuscript so that Fig. 2 (previously Fig.3) is explained more clearly and the definition of CT beams appears at the beginning of the "Experimental setup" subsection in the main text.

Comment 9

9. What is "the probability distribution of the mean number of detected photons?" Is it the distribution of the number of photons, or is it actually the distribution of the mean? Anyway, I assume it's the probability distribution for the number of photons; in which mode, then? Is it the two-mode joint distribution or the one-mode marginal distribution? Again I see an answer in the methods but the main text is confusing on its own.

Response 9

Thank you for this comment. By the probability distribution of the mean number of detected photons, we meant the probability distribution of the number of detected photons in the mode *d,* which is one mode marginal distribution, as under the experimental parameters, it is sufficient to measure the photons only in one of the modes. We have amended the text to clarify this part. We have also added the explanation at the beginning of the subsection "Field estimation via Rabi oscillations" in the body of the manuscript.

Comment 10

10. What is the expected advantage in FI given the toy model, assuming photon detection as the measuremet scheme? How does it compare to the 3.3 advantage here? Does the QFI indeed diminish by a factor of eta in this case, thereby justifying the normalization used to compute the 3.3 advantage? Otherwise unfortunately most of these comparisons are meaningless.

Response 10

Thank you for this comment. The toy model shows a twofold improvement compared to direct detection without the quantum channel \Lambda, as shown in Fig. 5d (previously Fig. 1d). However, we stress that the toy model serves only to demonstrate a simplified situation which leads to a desired behaviour, i.e. the increase of FI as a result of applying an operation before lossy readout. This toy model corresponds to our physical system, but in a slightly different regime of having only two excitations and a stronger interaction.
The full influence of the dipolar interaction between collective Rydberg excitations is more accurately described by the multi-particle model (previously phenomenological), considered in the "Methods", which corresponds better to the experimental situation. Those models are, however, fully compatible and simply describe different regimes.

In Figure 4b (previously Figure 5b), we compare the Fisher Information calculated from the multi-particle model (previously phenomenological).; the estimated values of \theta_ML are in good agreement with the set values of \theta (Fig. 4(c)), and the inverse of the variance of the estimated values of \theta matches the theoretically derived Fisher information (dashed line in Fig. 4(b)). Additionally, we have now added analytical derivations of the FI scaling under losses (with and without \Lambda) in the "Methods" in paragraph "Optimality of the pre-measurement

processing protocol". In the experiment, it is not possible to "remove" the losses to verify that QFI is diminished by a factor \eta. Instead, we directly compare a situation with and without the operation \Lambda to demonstrate the enhancement. Proper normalisation of photon numbers and matching to theory models provide a fair comparison.

Comment 11

11. What do the numbers mean when it says there is an average of 1.1 photons per pulse, the uncertainties are shown per 100 detected photons, and the estimation precision is per experimental shot; does this mean the precision of Delta E_MW is given per 100/1.1=91 pulses? Is this factored into the duty cycle calculation, then, by assuming some repetition rate of the pulses?

Response 11

Thank you for this question. By 1.1 photons per pulse, we understand that after the acquisition and detection of photons in the single experimental shot, we obtain, on average, 1.1 photons. We set the repetition rate of the experiments to 50 Hz to synchronise with the mains frequency.

In the article, we detect the photon statistics from the experiment, and we then calculate the maximum likelihood estimator and recover the variance of the estimator via the methods described in the "Field estimation via Rabi oscillations" paragraph. The value of the variance of the estimator is calculated per experimental shot (but the values on the plot are shown per N=100 measurements for clarity). From that, we calculate the sensitivity of the detector; we assume a 100% duty cycle, but we believe that a 50% duty cycle would be feasible, as stated at the end of the "Field estimation via Rabi oscillations" subsection.

Thank you for drawing our attention to this inconsistency. We revised the phrasing of the relevant values. Additionally, we noticed and fixed a misprint in the uncertainties in Fig. 4(a) (previously Fig. 5(a)), the uncertainties were shown per 100 shots, and we added the explanation to the text. We additionally expanded the section "Field estimation via Rabi oscillations" to include the detailed procedure of the estimation.

Comment 12

12. Now we get back to Fisher information per detected photon. This is actually the realm of postselected metrology and needs to be appropriately quantified. If you triple the FI/photon and reduce the probability of detecting a photon by 3 then you have not achieved anything overall, except in the case of detector saturation. So the advantage in the experiment is certainly not obvious.

Response 12

Thank you for this important remark. In this experiment, we are not using any post-selection method as the Reviewer mentioned before. As the Reviewer rightfully noticed, normally, the metrological advantage would fade away if we increased FI per photon and, at the same time, reduced the efficiency threefold. However, in the case of interactions, the FI does not scale linearly with efficiency, as it is visible in Figure 5(c) (previously 1(c)). This is exactly the reason

why, in the article, we compared the FI without interaction to the FI with interactions, as this allows an easy comparison of the two cases.

To benchmark the meteorological advantage in the experiment, we presented FI normalised by the value of FI without the interaction (F_wo = n_0 * \eta = \bar{n}=1.1). \bar{n} corresponds to the mean number of detected photons without interactions and for \theta=0. This way, we were able to easily compare the Fisher information per detected photon in both cases.
In the revised version of the article, we explicitly introduced a "normalised" FI (\tilde{\mathcal{F}}) to avoid further confusion (in paragraph "Field estimation via Rabi oscillations").
If we reduced the efficiency of the detection, leading to a smaller mean number of detected photons, we would still achieve an advantage over the protocol without the interaction. We have added the explanation to the article in the subsection "Field estimation via Rabi oscillations" so that it is clearer.
It is worth noting that we have chosen the regime with a significantly large \gamma so that we obtain the advantage in the experiment, despite additional losses. Choosing between regimes for the best improvement is depicted in Figure 9.

Comment 13
13. Getting to the methods, this does not seem to be sufficient for reproduceability. The authors claim that they used some optimization strategy defined in their previous work and then the numerically found optimal solution coincides with their toy model. The reader is expected to take all of this on faith and then also believe that the numerical solution is therefore the global optimum. It may all be true, but as a Reviewer I cannot accept any of it. There isn't even some analytical evidence that the current strategy is a local optimum of any sort.

Response 13
Thank you for this comment. In the revised version of the article, we included a link to the optimisation script used for determining the optimality of the operation. We also included an extended explanation of the bounds that the toy model saturates at the end of the "Toy model" paragraph. In the same paragraph, we also included an analytical formula for the FI with and without the operation \Lambda. Now, besides the numerical optimisation, we show that our results saturate bounds on FI given in PRX Quantum 4, 040305.

In the revised version of the "Methods", we included a section "Optimality of the pre-measurement processing protocol" which focuses on explaining the efficacy of the protocol, taking into account the theoretical results presented in other, related works. We also derive the analytical bounds given the assumed operation, and show that the toy model saturates the theoretical limit of the FI.

Comment 14
14. Then in the methods it appears that a single-photon counting module is used; again, this looks different from the theory of looking at the full photon number distribution or at states like |20> and |02>.

Response 14

Thank you for addressing this important point. The toy model was presented as a simple idea of the experiment, allowing for the demonstration of the meteorological gain by removing the state with overlapping decay paths from the detection. We do not claim that it fully describes the experimental setting. Please note that we store and measure coherent states, which have the Poissonian photon number statistics. In the scenario described by the multi-particle model (previously phenomenological), we detect the photons in the set window of time determined by the length of the readout impulses.
During this period, we measure the number of arriving photons corresponding to the stored excitations. Dead time of the used detectors is significantly smaller than the duration of the read-out impulse, meaning that we can measure the distribution of the number of photons just by repeating the experiment multiple times. Also, due to the very large losses in the experiment, the distribution of the arriving photons is very well described by the Poissonian statistics.

Furthermore, the measurement losses in the experiment are large, and on average, we measure ~1.1 photons per sequence, so the probability of detecting two photons is low (as it scales as \eta^2). As we detect the Poissonian statistics of photon distribution, we can estimate that the probability of two photons arriving is p(2) = 0.2 and p(3) = 0.07.

Comment 15
15. Still in the methods, now it looks as though the mean photon number in mode d is actually just computed as the mean photon number in mode d that arises from the |1,1> state. This is quite different from what was described in the main text. As well, nothing is given to explain how this evolution affects the other states, especially given that we have a pair of weak coherent states.

Response 15
Thank you for this important remark. In the Methods section, we describe the model for the two interacting Rydberg excitations resulting from dipolar interactions. This way, we can calculate the evolution of two interacting excitations, compute the decay rate, and extend the result for many excitations.
In this simple model for two interacting excitations, the evolutions for excitations in the mode *d* and *p* are independent, and for the states |2,0> and |0,2>, there are no interactions (V_dip = 0). Hence, we do not consider those states as they do not provide any information on the evolution under the influence of the interactions. The only non-trivial part comes from |1,1>.

However, in the experiment, we always have more excitations than 2, as we store the Rydberg excitations as coherent states. Due to the small number of initial excitations compared to the number of atoms, we can assume that there are only interactions between a pair of Rydberg excitations, and we can neglect many-body interactions. We included this explanation at the beginning of the "Generalisation to multi-particle interaction model" subsection in the Methods section.

The need to extend the model to include the coherent states is what motivated us to introduce a multi-particle model (previously phenomenological) in "Methods", which explains the evolution more accurately.

During the detection stage of the experiment, we measure the photons in the mode *d* and compute the average number of detected photons for each angle of Rabi rotation. When we place the initial state in a superposition between two Rydberg levels, the excitations begin to interact with the dipole potential, leading to dephasing of the stored excitations and effectively reducing the mean number of detected photons, as the phase-matching condition is no longer satisfied, all depending on the angle of rotation. The rate of decay of the mean number of photons in the mode *d* depends on the percentage of the population transferred to the mode *p,* as shown in Fig.4(d) (previously Fig.5(d)).

Comment 16

16. Finally, the phenomenological model for the photon statistics is fine, but I am not sure how seriously I should believe that it actually models the evolution (except perhaps in the case of very weak coherent states?).

Response 16

We appreciate the comment. We have also refined the multi-particle model (previously phenomenological), exploring two important regimes and showing the connection between the toy model and multi-particle and two-particle models. It is important to notice that, while for the \theta =0 and no interactions, we detect 1.1 photons on average due to efficiency, the number of stored Rydberg excitations is ~100 per sequence. We also want to work in a regime where there are no short-range van der Waals interactions between Rydberg atoms in the same state. This required us to induce spinwaves using weak coherent states, assuring a small number of Rydberg excitations and only long-range interactions. We have amended the last paragraph in the "Experimental setup" subsection to clarify this part.
To check whether our model correctly describes the evolution of the mean number of detected photons, we fitted a multi-particle model to the measured data for modes *d* and *p* as presented in Fig.3 (previously Fig.4). The fitted functions accurately describe the measured data. We also measured the decay rate of the stored Rydberg excitation as a function of the angle of the Rabi rotation and the Rydberg interaction time. Both results are consistent with the multi-particle model for the atoms in the coherent state.

Reviewer #2 (Remarks to the Author):

Comment 0
I co-reviewed this manuscript with one of the reviewers who provided the listed reports. This is part of the Nature Communications initiative to facilitate training in peer review and to provide appropriate recognition for Early Career Researchers who co-review manuscripts.

Response 0
We would like to thank the reviewer for contributing to refining the manuscript.

Reviewer #2 (Remarks on code availability):

Comment 1
I have able to install and run the numerical analysis code related to the optimality of the pre-measurement processing protocol. The authors have provided clear and well-documented instructions for installing and executing the code.

Response 1
We would like to thank the reviewer for testing the provided optimisation script.

Reviewer #3 (Remarks to the Author):
Comment 0
In the response letter by the authors, they "stress again that the main contribution of this paper is experimental and not theoretical, and the theory part served merely as a means to understand the naturally appearing `detection noise robustness' mechanism that we observed in the experiment." This complicates the question of whether the manuscript should be worthy of publication in Nature Communications, as the main work seemed to include much theory. One manner in which the authors have addressed this issue is by modifying the order of presentation so that the toy model appears last. This certainly helps centre the discussion on the experiment but I worry that it is a bait-and-switch tactic for the referees, who have mainly focused on the theory in the first round of review, and for the editors in prompting them to select theorists as reviewers.

Overall, I think it is nice to have an experiment showcasing that a state may be modified prior to an inefficient detector in order to restore some of the performance of the detector. I am not convinced that this method is general for overcoming loss in arbitrary platforms with arbitrary input states, but this demonstration is meritorious. I no longer feel strongly that this manuscript should be rejected from Nature Communications and am rather neutral about whether the experiment itself is worthy of publication there, with a slight leaning toward recommending publication due to this being am important theory (not novel here) that has not yet been demonstrated (to the best of my knowledge).

Response 0

We would like to thank the reviewer for helping us refine the manuscript. We would like to assure that it was not our intention to mislead the referees by changing the order in which we present the results. Initially, we thought that this order was more appealing for a broader audience, since starting from a simple model (which admittedly was only an approximation to the experimental setting) the manuscript took the readers to a more complex experimental implementation. Upon more consideration, we no longer feel this way and agree with reviewers' remarks.

During corrections following the reviewers' comments, we discovered a mistake, which slightly changed figures of merit in the manuscript. The affected results are: FI [3.6(3) -> 3.3(3)], normalized FI [3.3(3)->3.0(3)], \Delta E_MW [44uV/cm -> 217(8) uV/cm]. We would like to stress that this does not affect the conclusions and overall improvement in estimation precision. Additionally, following Reviewer #4 Comment 7, we included the script used for data analysis along with the deposited data in [56] (formerly [55]).

Comment 1

In response to the responses to referee #3, there are two outstanding points:

1. The language in the introduction is much more accurate and I appreciate the clarifications. However, it still just states that the QFI diminishes linearly in typical situations and does not provide justification/citation, which I feel may be necessary when this is the major hurdle surmounted by this work. Otherwise it is a straw man argument.

Response 1

We have now added a citation (section "Toy Model", [55]) to the work that explicitly states this - J. Kołodyński and R. Demkowicz-Dobrzański, New Journal of Physics 15, 073043 (2013). In Table 1, the first column, there is a list of values of Quantum Fisher Information that are achievable with independent probe strategies under different decoherence processes. In particular, there is a row describing the effects of loss, and the factor \eta appears there explicitly.

One can also use the following simple argument. If for N independent probes QFI is N F1, where F1 is a single probe QFI, then if the probability of loss of each probe is (1-\eta), then on average we are left with \eta N intact probes, each contributing F1 to the total QFI. Hence, the resulting QFI is reduced by a factor of \eta compared with the original one. We have added a footnote in the Methods section (section "Derivation of the FI for the toy model") with this argument.

Comment 2

2. I cannot find where the authors included non-linear amplification. Is this supposed to be subsumed by the Gamma in the Methods section Eq. (38)? The response 3 says that the scaling with losses would be "even more favourable" but the section surrounding Eq. (38) says that the present method is already optimal. What is the true result?

Response 2

Thank you for this remark. The testing of the amplification procedure is included in the script `nonlinear_amplification.py` in the deposited dataset (previously [55], now [56]).

In the included script, it is worth noting that the considered Hilbert space in `nonlinear_amplification.py` goes outside of the "Toy Model" assumptions. The amplification procedure requires extending the allowed excitation numbers to higher values.
The theoretical results presented in "Toy Model" apply to a simplified scenario, which is an approximation of the experimental setting. This assumes that the total number of excitations is at most 2, and the following numerical optimisation and the analytical derivation are limited to this space.

In case of amplification, the scaling is, in fact, more favourable (i.e. factor γ given by Eq. (40) scales differently with losses), but the exact form of the factor is outside the scope of this manuscript. The optimality of the assumed form of amplification lacks a formal proof; however, we believe that this numerical calculation (presented in the attached optimisation script) assures that there is hope for further improving the scaling. While its experimental implementation is not yet clear, the amplification procedure would be an interesting path to explore.

Reviewer #4 (Remarks to the Author):

Comment 0

The manuscript "Microwave-field quantum metrology with inherent robustness against detection losses enabled by Rydberg interactions" presents a clean and interesting experimental platform where intrinsic Rydberg-interaction-induced dynamics acts as a pre-measurement processing step, and the authors argue this can yield metrological robustness against imperfect detection. The story is attractive and the revision improves the narrative compared with the first round review. However, because the headline conclusion is quantitative (a reported multi-fold FI enhancement under loss), the paper should be held to a correspondingly quantitative standard: the FI extraction needs a statistically robust validation beyond a single estimator-variance route, the resource comparison must be explicitly apples-to-apples (per shot vs per detected photon vs per time), and the mechanism mapping between the effective channel picture and the actual experimental imperfections needs at least one discriminating diagnostic and a clearly stated validity window. If these items are addressed with minimal additional analysis (and possibly one simple control), I think the paper would become much more convincing.

After reading the revised manuscript and the authors rebuttal, I support both first-round referees as reasonable, because their start point and view point are different. One referee is more from conceptual novelty / theory framing side, another referee is more from rigor side: baseline definition, resource accounting, and theory–experiment correspondence. Both are fair.

Response 0

We would like to thank the reviewer for the response. We appreciate the in-depth analysis of our manuscript.

During corrections following the reviewers' comments, we discovered a mistake, which slightly changed figures of merit in the manuscript. The affected results are: FI [3.6(3) -> 3.3(3)], normalized FI [3.3(3)->3.0(3)], \Delta E_MW [44uV/cm -> 217(8) uV/cm]. We would like to stress that this does not affect the conclusions and overall improvement in estimation precision.

Additionally, following Reviewer #4 Comment 7, we included the script used for data analysis along with the deposited data in [56] (formerly [55]).

Comment 1

The central experimental claim relies on extracting FI from the variance of an estimator (MLE) with finite sample size per setting. In general, using $F \approx 1/(N\ \mathrm{Var}(\hat{\theta}))$ is asymptotic, but at $N \sim 100$ it can have bias or model-mismatch sensitivity. This matters because the headline enhancement factor can shift if finite-sample effects are not controlled. Provide an estimator-independent FI cross-check computed from measured outcome frequencies, or from a validated likelihood model with goodness-of-fit and uncertainty propagation. Give confidence intervals on the enhancement factor by a full pipeline bootstrap (fit → likelihood → estimator distribution → FI). State clearly what is the measurement outcome used in likelihood (only n_d ? or joint outcomes ?), and justify sufficiency.

Response 1

Thank you for this remark. To cross-check the validity of the results for our estimator, we have calculated the theoretical Fisher information using the parameters with uncertainties obtained from the model fitted to the collected data. The enhancement factor (normalised FI) in this case is \tilde{F}=3.0±0.3. We also want to stress that the precision of the estimation \Delta^2 \theta was bootstrapped for different divisions of k and N, where k*N=10000 (i.e. [100,100],[200,50], etc), where the nominal value and error were calculated as the mean and standard deviation taken from different divisions.

In Figure 4(a), the variance was presented per N=100 experimental shots only for clarity of the plot, so that the error bars do not cover the entire subfigure. We included the propagated error from the goodness-of-fit of the decay rate (\gamma) as shaded areas in the subfigure **b**.

For the maximum likelihood estimation, we use the probability distribution n_d (from Eq. (26)), which is our measurement outcome, and the model fitted to the mean number of detected photons is the mean number of photons calculated from these statistics. In the included reproducibility script (deposited data, previously [55], now [56]), we show the analysis leading up to the presented figures of merit in the manuscript.

Furthermore, to validate the asymptotic behaviour of MLE in our case, we used a Monte Carlo simulation to generate synthetic data according to the probability given in Eq. (26). We generated N samples, then calculated the ML estimator and repeated this process N_rep = 400 times for each N. The resulting estimate, variance (\Delta^2 \theta) and 1/(N\Delta^2 \theta) are plotted below. We see that even for sample size N=50, the variance approaches the asymptotic value. Here, the errorbars for the mean are calculated as the standard deviation of the mean (\sqrt{\Delta^2 \theta / N_{rep}}) and for variance, it is the square root of mean squared error (\Delta^2\theta \sqrt{2/(N_{rep}-1)}).

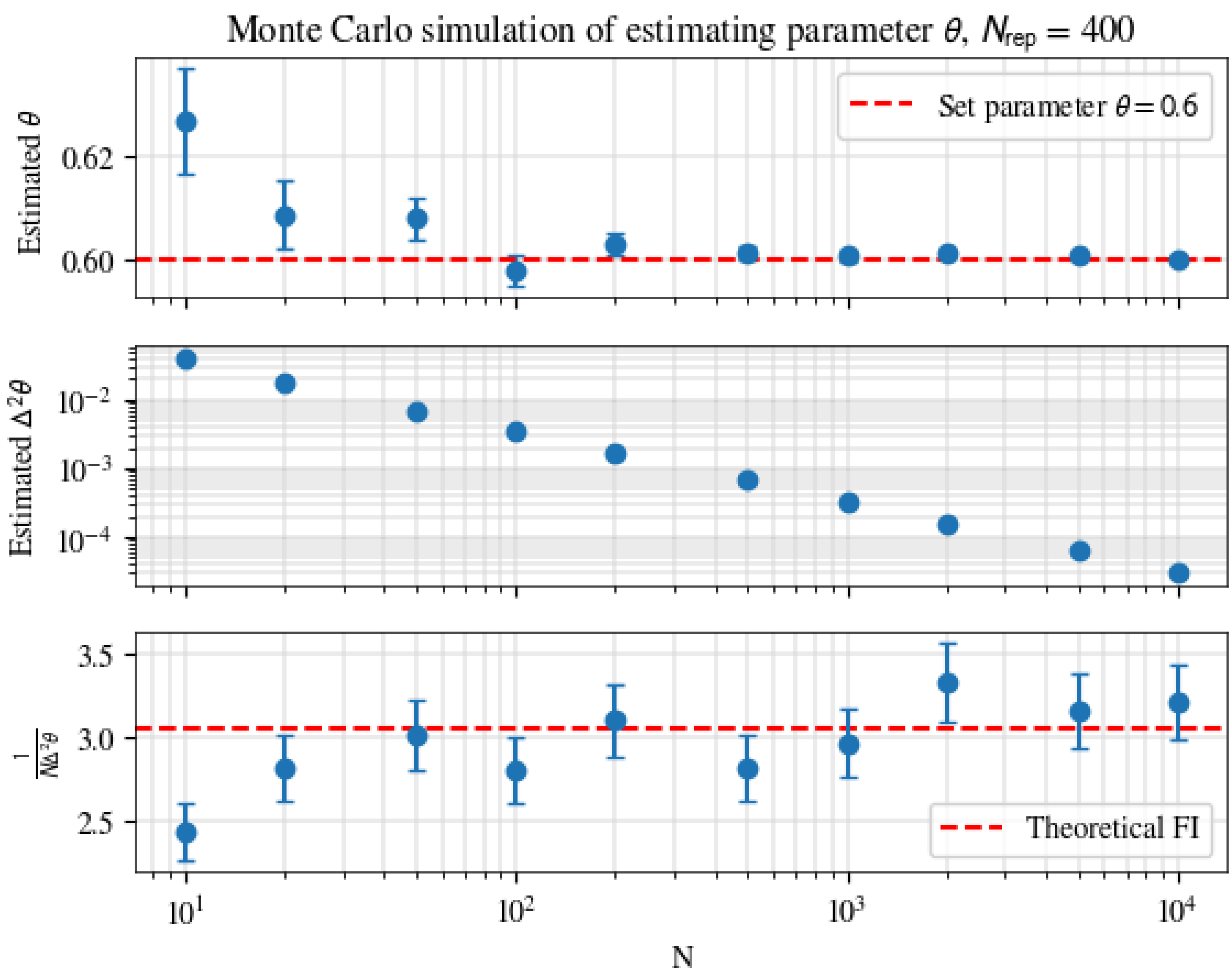

Comment 2

The manuscript uses a normalized FI (roughly $F/\bar{n}$) which is meaningful as "information per detected photon". But the preprocessing also changes detection statistics, so normalization can hide trade-offs. Readers need to see clearly what is improved under what constraint. Add a small table listing key parameters at the operating point(s): cycle time, sensing time, overhead, τ , mean detected counts $\bar{n}$ with/without interactions, and detection window. Show both $F(\theta)$ per shot and $F(\theta)/\bar{n}$ on the same dataset (with uncertainties). Clearly define the baseline constraint: fixed prepared excitations? fixed mean detected photons? fixed total time?

Response 2

We would like to thank the reviewer for this important comment. We would like to point out that the factor used for normalization of Fisher Information (\bar{n}) is the same for each oscillation angle \theta. This factor is measured once and corresponds to the detected number of photons without interactions (i.e. for \theta = 0). We fully agree that the preprocessing changes the number of detected photons, however the presented normalization is not influenced by this.

Note that \bar{n} = \eta n_0, which is the initial FI (without interactions, i.e., maximal obtainable for measuring Rabi oscillations *without preprocessing*). FI normalised this way shows the true enhancement of the method compared to the situation without preprocessing (regardless of the number of detected photons at the operating point). As we stressed in the previous response (Reviewer #3 Response 4 and Response 12), we do not exceed FI *without losses*, as this would be impossible; however, because the preprocessing happens before losses, our state is more resilient to information loss (scales more favourably than \propto \eta).

We changed the problematic phrasing "per detected photon" to "normalised" to avoid confusion. We included the implicit assumptions regarding the validity of normalised FI below Eq. (8). Additionally, we included a table (in subsection "Field estimation via Rabi oscillations") comparing the expected values of metrics without preprocessing with the same number of initial excitations and detection efficiency. We included the table with key parameters used in the operating point in the Methods section.

Comment 3

The regulated-loss channel picture is plausible, but other effects can mimic decay (technical phase noise, inhomogeneous dephasing, drift). For a broad audience, it is important to show at least one discriminating signature specific to the proposed mechanism, not only exponential fitting. Add a concise "validity conditions" paragraph: regime of $\gamma\tau$, pair-dominance vs many- body, assumptions behind Poisson-mixture model, etc. Provide one discriminating scaling check (e.g., vary n_0, density/OD, or τ) and show extracted decay rate and/or FI enhancement follows the model trend.

Response 3

Thank you for this important remark. In the derivation of the probability distribution in Eq. (26), we assume that the only interactions between the Rydberg atoms are two-particle interactions, and we do not consider multi-particle interactions and any additional many-body effects. Additionally, we assume \gamma\tau << 1, which is the correct regime for our experiment, since from the data we measured \gamma\tau to be approximately 0.04.

For the Poissonian mixture model, we assume that:

- The distribution of the number of excitations in both modes follows the coherent state statistics
- The losses in one mode only depend on the number of excitations in the opposite mode

We included the assumed conditions in the "Field estimation via Rabi oscillations" section.

Additionally, we have measured the super-rabi oscillation for different interaction times and extracted the decay rate $\gamma\tau$, which increases linearly with the interaction time, showing that it follows the theoretically predicted results. We added this result as the subfig. **f** in Figure 3.

Comment 4

The narrative focuses on detection efficiency η, but realistic photon counting also has back-ground/dark counts and dead time. A reshaped photon-number distribution can change sensitivity to these non-idealities. This can be a hidden systematic in FI. Add quantitative bounds: dark count rate vs detection window, background subtraction method, dead time relevance (multi-click probability). If available, include a "no-signal" histogram or background measurement in SI.

Response 4

Thank you for this question. In our experiment, we used a single-photon counting module (SPCM-AQRH-14, Excelitas) with a dead time of 22 ns and a dark count rate of 100 counts/s, which corresponds to a background noise of 0.008 photons/sequence/window for the same detection window used to collect photons for the MW field estimation. The detection window is

900ns, which is much longer than the detector dead time. We do not subtract the background from the data used in the analysis. For this detection window, we measure 1.1 photons/sequence for the \theta=0. As we detect the Poissonian statistics of photon distribution, we can estimate that the probability of two photons arriving is p(2) = 0.2 and p(3) = 0.07, so the probability of detecting multiple clicks in one bin is low. We have added the histogram and the number of collected counts in the detection window used in the experiment for the 'no signal' measurement as the Extended data figure 10.

Comment 5

The conversion from an estimated angle (or Rabi frequency) uncertainty to field sensitivity and then to $\mathrm{Hz}^{-1/2}$ depends on timing assumptions. The text should let readers reproduce the number without guessing. Add a short bookkeeping paragraph: define θ, sensing time T, cycle time, duty cycle used, and show the arithmetic for the quoted $\mathrm{Hz}^{-1/2}$ sensitivity. Provide realistic range (e.g., 50% duty cycle) as well as ideal 100% assumption, clearly labeled.

Response 5

Thank you for this important comment. In the previous version of the manuscript, we calculated the MW field sensitivity by taking the minimal field variance (\Delta E_{MW}=217uV/cm) and assuming that the repetition rate of the measurement is the duration of the MW pulse (T=160ns, with current parameters the value would be ~87nV/cm/\sqrt{Hz}).

We argue that reasonable performance could be achieved with a duty cycle of 50%. The 50% duty cycle can be achieved, for instance, by performing multiple experiments in one sequence. Atoms under the investigation can be loaded into the optical dipole trap with the trap lifetime longer than half the time of the sequence, allowing for multiple repetitions of the same experiment.

The comment motivated us to reconsider how we calculated the figure of merit used to define the MW field sensitivity of the presented protocol. In our case, sensitivity S_{E_{MW}} strongly depends on the duration of a single experiment, making it unsuitable for characterising the sensor's detection capabilities. Instead, we opted for a different metric, \bar{S} = \Delta E * *T*, which is now independent of the experiment duration and enables us to anticipate the ultimate limits of the proposed method.

To consider the limits of this scheme, we assumed that in the optimal case, the duration of the pulse is as long as possible. That said, the limiting factor is the coherence time \tau_{mod} = 31 us. This way, we can define the ultimate achievable sensitivity as \tilde{S}/sqrt(\tau) = 6.3 nV/cm/\sqrt{Hz}. This assumes that the measurements are taken continuously (for example, with the previously mentioned subsequent experiments in a single dipole trap).

We removed from the manuscript the discussion considering the duty cycle of the protocol, and expanded the discussion regarding the more suitable figure of merit. We sincerely hope that this new expanded approach is better suited to the physical reality, as well as more adjusted to trigger future analyses by other researchers.

Comment 6

The advantage likely holds in a window (small excitation number, moderate $\gamma\tau$, not too strong dephasing). It is important to state where it works and where it fails, otherwise readers may over-interpret. Add a boundary plot or table (can be semi-quantitative) showing enhancement vs ($\gamma\tau$, $\bar{n}$) or vs (τ, n_0). Add explicit scope statements: what happens at larger photon number, higher density, longer τ .

Response 6

Thank you for this comment. A larger number of photons leads to an increase in the Fisher information as long as $\gamma > 0$. For small numbers of photons, there is a region for which the Fisher information is decreased compared to the no-interaction regime. This is depicted in Fig. 9a in the Methods section.

The longer times (larger $\gamma\tau$ product) for a given number of atoms, the Fisher information at first decreases and then increases up to the maximal value for which it saturates, and further increase of $\gamma\tau$ product does not improve the Fisher information per detected photon. Atomic density does not directly contribute to the enhancement in the Fisher information, although it affects the storage and readout efficiency, which affects the overall efficiency of the protocol.

We added the "boundary plot" (γτ,n_0) showing the maximal Fisher information for the optimal \theta as the Extended data Figure 9c in the Methods section.

Comment 7

Minor comments:

- Define η, η_{det}, n_0, $\bar{n}$ in one place; keep consistent notation.
- Avoid calling something "classical limit" if it is a model-specific baseline; rename as "standard no-preprocessing scaling in this setting".
- Explicitly state the likelihood model and the measured outcome used.
- In Fig. 4 caption: specify N used, number of bootstrap resamples, and what is randomized.
- Provide the exact parameter set for the reported 3.3 factor point: τ , extracted γ, n_0, $\bar{n}$, MW pulse time, detection window.
- State clearly whether any trials are discarded (loading failure, detector glitch). If yes, fraction and impact.
- Clarify whether γ is independently calibrated or jointly fitted; if joint, report correlations.
- For $Hz^{-1/2}$ sensitivity: state the cycle time used and whether 12 ms is full cycle.
- Provide a minimal reproducibility note: data/code location, script version, inputs/outputs.
- Move the most important validity assumptions from deep Methods into main Discussion.

Overall I think the idea and experiment can be publishable. The revision improved clarity compared to *the first* round, but I still see several points that can decide acceptance quality.

Response 7

We updated the section of the manuscript where \eta_\text{det} appears. We defined the important variables in the section 'Theoretical Model', below Eq. (5).

We replaced the confusing ‘classical’ with the proposed, more precise alternative.

We expanded the explanation regarding the probability used in maximum likelihood estimation.

We expanded the caption of Fig.4 by directly stating the number of shots used for each point and the number of resamples used in the bootstrapping.

We included the exact values of the parameters used in calculations in the text.

We stress that all the measured values were included in the analysis; we did not post-select the results. We included an additional explanation regarding this.

γ was measured independently, separately from the super Rabi oscillation method. The results are shown in Fig. 3b; the decay measurement protocol is shown in Fig. 3a. We fitted an exponential decay function (A*exp(-\gamma\tau)) for each of the oscillation angles (durations of the MW pulses), and the resulting decay rates, along with the fit uncertainties, are shown in Fig. 3c.

We reworked the sensitivity section to include figures of merit more appropriate for this protocol.

We included a reproducibility script, along with the ML estimation (for Figure 4.), in the deposited data (previously [55], now [56])

We included the assumptions used in the derivation in the section “Field estimation via Rabi oscillations”.

Dear Editor
Thank you for considering our work and extending a publication offer for our manuscript. We shall repeat the Reviewer's comments in red while responding in blue.

on behalf of all the authors
M. Parniak

REVIEWERS' COMMENTS

Reviewer #3 (Remarks to the Author):

Comment 1
The authors have adequately addressed most of my queries. I am not certain why most of the amplification material is in a python script in the attached dataset and believe that explaining this in more detail in the main text would be reasonable.

Response 1
Thank you for this comment. We added a brief explanation of the amplification procedure in the last part of the "Discussion" section. We believed that a lack of formal proof reduced the appeal of this approach; however, after the reviewer's comments, we decided to include it nonetheless.

Reviewer #3 (Remarks on code availability):

n/a

Reviewer #4 (Remarks to the Author):

Comment 0
I have carefully examined the revised manuscript and the authors' response to my previous comments. Overall, the authors have addressed the major concerns in a careful and largely satisfactory manner. In particular, the Fisher-information analysis has been strengthened by including a model-based FI cross-check with propagated uncertainties, an explicit statement of the likelihood outcome nd, bootstrapping of the estimator variance, and an additional Monte Carlo validation of the MLE behaviour. The resource comparison has also been clarified by replacing the potentially confusing "per detected photon" wording with a more precise normalised-FI discussion and by adding the relevant operating parameters.
I am especially encouraged by the new diagnostics for the physical mechanism. The added τ-dependent scaling check, the stated validity conditions such as $\gamma\tau \ll 1$, the discussion of pair-dominance assumptions, and the boundary plot in the Methods go beyond the minimum needed to address my original concern. The quantitative treatment of dark counts, dead time, and the no-signal background histogram also makes the experimental claim more credible. One minor caveat remains: the FI cross-check is still primarily model-based rather than a fully estimator-independent reconstruction directly from raw outcome frequencies. However, given

the additional uncertainty propagation, bootstrap analysis, and Monte Carlo validation, I do not think this warrants another review round.
I therefore find the manuscript substantially improved and suitable for publication after minor editorial corrections.

Response 0
Thank you for finding our work worthy of publishing in *Nature Communications*.

Comment 1
A few small typos in the main text should be corrected:
- "This effect has been exploited ..., but have never been demonstrated" → "..., but has never been demonstrated".
- In the caption of Fig. 1, the panel label "(d)" appears twice; the second occurrence should likely be "(e)" (in my opinion).
- Sentences beginning with "Where ..." immediately after equations should use lowercase "where".
- "precision achieved per a single shot" should be "precision achieved per single shot" or "precision achieved in a single shot".

Subject to these minor textual corrections, I support acceptance of the manuscript in Nature Communications.

Response 1
We sincerely thank the reviewer for their exceptionally careful reading of our manuscript. We have corrected the typos in the main text and figure captions.

The manuscript "Microwave-field quantum metrology with inherent robustness against detection losses enabled by Rydberg interactions" presents a clean and interesting experimental platform where intrinsic Rydberg-interaction-induced dynamics acts as a pre-measurement processing step, and the authors argue this can yield metrological robustness against imperfect detection. The story is attractive and the revision improves the narrative compared with the first round review. However, because the headline conclusion is quantitative (a reported multi-fold FI enhancement under loss), the paper should be held to a correspondingly quantitative standard: the FI extraction needs a statistically robust validation beyond a single estimator-variance route, the resource comparison must be explicitly apples-to-apples (per shot vs per detected photon vs per time), and the mechanism mapping between the effective channel picture and the actual experimental imperfections needs at least one discriminating diagnostic and a clearly stated validity window. If these items are addressed with minimal additional analysis (and possibly one simple control), I think the paper would become much more convincing.

After reading the revised manuscript and the authors rebuttal, I support both first-round referees as reasonable, because their start point and view point are different. One referee is more from conceptual novelty / theory framing side, another referee is more from rigor side: baseline definition, resource accounting, and theory–experiment correspondence. Both are fair.

The central experimental claim relies on extracting FI from the variance of an estimator (MLE) with finite sample size per setting. In general, using $F \approx 1/(N\,\mathrm{Var}(\hat{\theta}))$ is asymptotic, but at $N \sim 100$ it can have bias or model-mismatch sensitivity. This matters because the headline enhancement factor can shift if finite-sample effects are not controlled. Provide an estimator-independent FI cross-check computed from measured outcome frequencies, or from a validated likelihood model with goodness-of-fit and uncertainty propagation. Give confidence intervals on the enhancement factor by a full pipeline bootstrap (fit $\rightarrow$ likelihood $\rightarrow$ estimator distribution $\rightarrow$ FI). State clearly what is the measurement outcome used in likelihood (only n_d ? or joint outcomes ?), and justify sufficiency.

The manuscript uses a normalized FI (roughly $F/\bar{n}$) which is meaningful as "information per detected photon". But the preprocessing also changes detection statistics, so normalization can hide trade-offs. Readers need to see clearly what is improved under what constraint. Add a small table listing key parameters at the operating point(s): cycle time, sensing time, overhead, τ, mean detected counts $\bar{n}$ with/without interactions, and detection window. Show both $F(\theta)$ per shot and $F(\theta)/\bar{n}$ on the same dataset (with uncertainties). Clearly define the baseline constraint: fixed prepared excitations? fixed mean detected photons? fixed total time?

The regulated-loss channel picture is plausible, but other effects can mimic decay (technical phase noise, inhomogeneous dephasing, drift). For a broad audience, it is important to show at least one discriminating signature specific to the proposed mechanism, not only exponential fitting. Add a concise "validity conditions" paragraph: regime of $\gamma\tau$, pair-dominance vs many-body, assumptions behind Poisson-mixture model, etc. Provide one discriminating scaling check (e.g., vary n_0, density/OD, or τ) and show extracted decay rate and/or FI enhancement follows the model trend.

The narrative focuses on detection efficiency η, but realistic photon counting also has background/dark counts and dead time. A reshaped photon-number distribution can change sensitivity to these non-idealities. This can be a hidden systematic in FI. Add quantitative bounds: dark count rate vs detection window, background subtraction method, dead time relevance (multi-click probability). If available, include a "no-signal" histogram or background measurement in SI.

The conversion from an estimated angle (or Rabi frequency) uncertainty to field sensitivity and then to $\mathrm{Hz}^{-1/2}$ depends on timing assumptions. The text should let readers reproduce the number without guessing. Add a short bookkeeping paragraph: define θ, sensing time T, cycle time, duty

cycle used, and show the arithmetic for the quoted $\mathrm{Hz}^{-1/2}$ sensitivity. Provide realistic range (e.g., 50% duty cycle) as well as ideal 100% assumption, clearly labeled.

The advantage likely holds in a window (small excitation number, moderate $\gamma\tau$, not too strong dephasing). It is important to state where it works and where it fails, otherwise readers may over-interpret. Add a boundary plot or table (can be semi-quantitative) showing enhancement vs $(\gamma\tau, \bar{n})$ or vs (τ, n_0). Add explicit scope statements: what happens at larger photon number, higher density, longer τ.

Minor comments

- Define η, η_{det}, n_0, $\bar{n}$ in one place; keep consistent notation.

- Avoid calling something "classical limit" if it is a model-specific baseline; rename as "standard no-preprocessing scaling in this setting".

- Explicitly state the likelihood model and the measured outcome used.

- In Fig. 4 caption: specify N used, number of bootstrap resamples, and what is randomized.

- Provide the exact parameter set for the reported 3.3 factor point: τ, extracted γ, n_0, $\bar{n}$, MW pulse time, detection window.

- State clearly whether any trials are discarded (loading failure, detector glitch). If yes, fraction and impact.

- Clarify whether γ is independently calibrated or jointly fitted; if joint, report correlations.

- For $\mathrm{Hz}^{-1/2}$ sensitivity: state the cycle time used and whether 12 ms is full cycle.

- Provide a minimal reproducibility note: data/code location, script version, inputs/outputs.

- Move the most important validity assumptions from deep Methods into main Discussion.

Overall I think the idea and experiment can be publishable. The revision improved clarity compared to the first round, but I still see several points that can decide acceptance quality.

I have carefully examined the revised manuscript and the authors' response to my previous comments. Overall, the authors have addressed the major concerns in a careful and largely satisfactory manner. In particular, the Fisher-information analysis has been strengthened by including a model-based FI cross-check with propagated uncertainties, an explicit statement of the likelihood outcome n_d, bootstrapping of the estimator variance, and an additional Monte Carlo validation of the MLE behaviour. The resource comparison has also been clarified by replacing the potentially confusing "per detected photon" wording with a more precise normalised-FI discussion and by adding the relevant operating parameters.

I am especially encouraged by the new diagnostics for the physical mechanism. The added τ-dependent scaling check, the stated validity conditions such as $\gamma\tau \ll 1$, the discussion of pair-dominance assumptions, and the boundary plot in the Methods go beyond the minimum needed to address my original concern. The quantitative treatment of dark counts, dead time, and the no-signal background histogram also makes the experimental claim more credible. One minor caveat remains: the FI cross-check is still primarily model-based rather than a fully estimator-independent reconstruction directly from raw outcome frequencies. However, given the additional uncertainty propagation, bootstrap analysis, and Monte Carlo validation, I do not think this warrants another review round.

I therefore find the manuscript substantially improved and suitable for publication after minor editorial corrections. A few small typos in the main text should be corrected:

- "This effect has been exploited ..., but have never been demonstrated" → "..., but has never been demonstrated".
- In the caption of Fig. 1, the panel label "(d)" appears twice; the second occurrence should likely be "(e)" (in my opinion).
- Sentences beginning with "Where ..." immediately after equations should use lowercase "where".
- "precision achieved per a single shot" should be "precision achieved per single shot" or "precision achieved in a single shot".

Subject to these minor textual corrections, I support acceptance of the manuscript in Nature Communications.
